# Connecting Deep Neural Networks to Physical, Perceptual, and Electrophysiological Auditory Signals

**DOI:** 10.3389/fnins.2018.00532

**Published:** 2018-08-14

**Authors:** Nicholas Huang, Malcolm Slaney, Mounya Elhilali

**Affiliations:** ^1^Laboratory for Computational Audio Perception, Department of Electrical and Computer Engineering, Johns Hopkins University, Baltimore, MD, United States; ^2^Machine Hearing, Google AI, Google (United States), Mountain View, CA, United States

**Keywords:** convolutional neural network, auditory salience, natural scenes, audio classification, electroencephalography, deep learning

## Abstract

Deep neural networks have been recently shown to capture intricate information transformation of signals from the sensory profiles to semantic representations that facilitate recognition or discrimination of complex stimuli. In this vein, convolutional neural networks (CNNs) have been used very successfully in image and audio classification. Designed to imitate the hierarchical structure of the nervous system, CNNs reflect activation with increasing degrees of complexity that transform the incoming signal onto object-level representations. In this work, we employ a CNN trained for large-scale audio object classification to gain insights about the contribution of various audio representations that guide sound perception. The analysis contrasts activation of different layers of a CNN with acoustic features extracted directly from the scenes, perceptual salience obtained from behavioral responses of human listeners, as well as neural oscillations recorded by electroencephalography (EEG) in response to the same natural scenes. All three measures are tightly linked quantities believed to guide percepts of salience and object formation when listening to complex scenes. The results paint a picture of the intricate interplay between low-level and object-level representations in guiding auditory salience that is very much dependent on context and sound category.

## Introduction

Over the past few years, convolutional neural networks (CNNs) have revolutionized machine perception, particularly in the domains of image understanding, speech and audio recognition, and multimedia analytics (Krizhevsky et al., 2012; Karpathy et al., 2014; Cai and Xia, 2015; Simonyan and Zisserman, 2015; He et al., 2016; Hershey et al., 2017; Poria et al., 2017). A CNN is a form of a deep neural network (DNN) where most of the computation are done with trainable kernel that are slid over the entire input. These networks implement hierarchical architectures that mimic the biological structure of the human sensory system. They are organized in a series of processing layers that perform different transformations of the incoming signal, hence “learning” information in a distributed topology. CNNs specifically include convolutional layers which contain units that are connected only to a small region of the previous layer. By constraining the selectivity of units in these layers, nodes in the network have emergent “receptive fields,” allowing them to learn from local information in the input and structure processing in a distributed way; much like neurons in the brain have receptive fields with localized connectivity organized in topographic maps that afford powerful scalability and flexibility in computing. This localized processing is often complemented with fully connected layers which integrate transformations learned across earlier layers, hence incorporating information about content and context and completing the mapping from the signal domain (e.g., pixels, acoustic waveforms) to a more semantic representation.

As with all DNNs, CNNs rely on vast amounts of data to train the large number of parameters and complex architecture of these networks. CNNs have been more widely used in a variety of computer vision tasks for which large datasets have been compiled (Goodfellow et al., 2016). In contrast, due to limited data, audio classification has only recently been able to take advantage of the remarkable learning capability of CNNs. Recent interests in audio data curation have made available a large collection of millions of YouTube videos which were used to train CNNs for audio classification with remarkable performance (Hershey et al., 2017; Jansen et al., 2017). These networks offer a powerful platform to gain better insights on the characteristics of natural soundscapes. The current study aims to use this CNN platform to elucidate the characteristics of everyday sound events that influence their acoustic properties, their salience (i.e., how well they “stand-out” for a listener), and the neural oscillation signatures that they elicit. All three measures are very closely tied together and play a crucial role in guiding our perception of sounds.

Given the parallels between the architecture of a CNN and the brain structures from lower or higher cortical areas, the current work uses the CNN as a springboard to examine the granularity of representations of acoustic scenes as reflected in their acoustic profiles, evoked neural oscillations, and crucially their underlying salience; this latter being a more abstract attribute that is largely ill-defined in terms of its neural underpinnings and perceptual correlates. Salience is a characteristic of a sensory stimulus that makes it attract our attention regardless of where our intentions are. It is what allows a phone ringing to distract us while we are intently in the midst of a conversation. As such, it is a critical component of the attentional system that draws our attention toward potentially relevant stimuli.

Studies of salience have mostly flourished in the visual literature, which benefited from a wealth of image and video datasets as well as powerful behavioral, neural, and computational tools to explore characteristics of visual salience. The study of salience in audition has been limited both by lack of data as well as limitations in existing tools that afford exploring auditory salience in a more natural and unconstrained way. A large body of work has explored aspects of auditory salience by employing artificially constructed stimuli, such as tone and noise tokens (Elhilali et al., 2009; Duangudom and Anderson, 2013). When natural sounds are used, they are often only short snippets that are either played alone or pieced together (Kayser et al., 2005; Duangudom and Anderson, 2007; Kaya and Elhilali, 2014; Tordini et al., 2015; Petsas et al., 2016). Such manipulations limit the understanding of effects of salience in a more natural setting, which must take into account contextual cues as well as complexities of listening in everyday environments.

Despite the use of constrained or artificial settings, studies of auditory salience have shed light on the role of the acoustic profile of a sound event in determining its salience. Loudness is a natural predominant feature, but is complemented by other acoustic attributes, most notably sound roughness and changes in pitch (Nostl et al., 2012; Arnal et al., 2015). Still, the relative contribution of these various cues and their linear or non-linear interactions have been reported to be very important (Kaya and Elhilali, 2014; Tordini et al., 2015) or sometimes provide little benefit (Kim et al., 2014) to determining the salience of a sound event depending on the stimulus structure, its context, and the task at hand. Unfortunately, a complete model of auditory salience that can account for these various facets of auditory salience has not yet been developed. Importantly, studies of auditory salience using very busy and unconstrained soundscapes highlight the limitations of explaining behavioral reports of salience using only basic acoustic features (Huang and Elhilali, 2017). By all accounts, auditory salience is likely a multifaceted process that not only encompasses the acoustic characteristics of the event itself, but is shaped by the preceding acoustic context, the semantic profile of the scene as well as built-in expectation both from short-term and long-term memory, much in line with processes that guide visual salience especially in natural scenes (Treue, 2003; Wolfe and Horowitz, 2004; Veale et al., 2017).

Convolutional neural networks offer a powerful platform to shed light on these various aspects of a natural soundscape and hence can provide insight into the various factors at play in auditory salience in everyday soundscapes. In the present work, we leverage access to a recently published database of natural sounds for which behavioral and neural salience measures are available (Huang and Elhilali, 2017, 2018) to ask the question: how well does activity in a large-scale DNN at various points in the network correlate with these measures? Owing to the complexity of these convolutional models, we do not expect an explicit account of exact factors or processes that determine salience. Rather, we examine the contribution of peripheral vs. deeper layers in the network to explore contributions of different factors along the continuum from simple acoustic features to more complex representations, and ultimately to semantic-level embeddings that reflect sound classes. A number of studies have argued for a direct correspondence between the hierarchy in the primate visual system and layers of deep CNNs (Kriegeskorte, 2015; Yamins and DiCarlo, 2016; Kuzovkin et al., 2017). A recent fMRI study has also shown evidence that a hierarchical structure arises in a sound classification CNN, revealing an organization analogous to that of human auditory cortex (Kell et al., 2018). In the same vein, we explore how well activations at different layers in an audio CNN explain acoustic features, behaviorally measured salience, and neural responses corresponding to a set of complex natural scenes. These signals are all related (but not limited) to salience, and as such this comparison reveals the likely contribution of early vs. higher cortical areas in guiding judgments of auditory salience.

This paper is organized as follows. First, the material and methods employed are presented. This next section describes the database used, the acoustic analysis of audio features in the dataset, and the behavioral and neural responses for this same set obtained from human subjects. The architecture of the neural network is also described as the platform that guides the analysis of other metrics. The results present the information gleaned from the CNN about its representation of acoustic, behavioral, and neural correlates of salience. Finally, the discussion section summarizes the insights gained from these results and its impact for future work to better understand auditory salience and its role in our perception of sounds.

## Materials and Methods

This next section describes the acoustic data, three types of auditory descriptors [acoustic features, a behavioral measure, and electroencephalography (EEG)], as well as three types of analyses employed in this study (CNN, surprisal, and correlation).

### Stimuli

The stimuli used in the present study consist of 20 natural scenes taken from the JHU DNSS (Dichotic Natural Salience Soundscapes) Database (Huang and Elhilali, 2017). Scenes are approximately 2 min in length each and sampled at 22,050 Hz. These scenes originate from several sources, including YouTube, FreeSound, and the BBC Sound Effects Library. The scenes encompass a wide variety of settings and sound objects, as well as a range of sound densities. Stimuli are manually divided into two groups for further analysis; a “sparse” group, which includes scenes with relatively few but clearly isolated acoustic events. An example of a sparse scene includes a recording of a bowling alley in which a relatively silent background is punctuated by the sound of a bowling ball first striking the floor and then the pins. The remaining scenes are categorized as “dense” scenes. Examples of these scenes include a maternity ward, a protest on the streets, and a dog park with continuously ongoing sounds and raucous backgrounds. This comparison between sparse and dense scenes is important because salience in dense scenes is particularly difficult to explain using only acoustic features, and thus more complex information such as sound category may provide a benefit.

### Acoustic Features

Each of the scenes in the JHU DNSS database is analyzed to extract an array of acoustic features, including loudness, brightness, bandwidth, spectral flatness, spectral irregularity, pitch, harmonicity, modulations in the temporal domain (rate), and modulations in the frequency domain (scale). Details of these feature calculations can be found elsewhere (Huang and Elhilali, 2017). In addition, the current study also includes an explicit measure of roughness as one of the acoustic features of interest. It is defined as the average magnitude of temporal modulations between 30 and 150 Hz, normalized by the root-mean-squared energy of the acoustic signal, following the method proposed by Arnal et al. (2015).

### Behavioral Salience

The Huang and Elhilali (2017) study collected a behavioral estimate of salience in each of the scenes in the JHU DNSS dataset. Briefly, subjects listen to two scenes presented simultaneously in a dichotic fashion (one presented to each ear). Subjects are instructed to use a computer mouse to indicate which scene they are focusing on at any given time. Salience is defined as the percentage of subjects that attend to a scene when compared to all other scenes, as a function of time.

Peaks in the derivative of the salience curve for each scene define onsets of *salient events*. These are moments in which a percentage of subjects concurrently begin listening to the associated scene, regardless of the content of the opposing scene playing in their other ear. The strength of an event is defined as a linear combination of the height of the slope at that point in time and the maximum percentage of subjects simultaneously attending to the scene within a 4-s window following the event. The strongest 50% of these events are used in the event-related analysis in the current study. These events are further manually categorized into one of seven sound classes (speech, music, other vocalization, animal, device/vehicle, tapping/striking, and other). The speech, music, other vocalization, vehicle/device, and tapping/striking classes contained the most number of events and are included in the current study for further analysis. By this definition of salience, the scenes contained 47 events in the speech class, 57 events in music, 39 events in other vocalization, 44 events in vehicle/device, and 28 events in tapping. The two remaining classes consisted of too few instances, with only 11 events in the animal category and eight in a miscellaneous category.

### Electroencephalography

Cortical activity while listening to the JHU DNSS stimuli is also measured using EEG, following procedures described in the study by Huang and Elhilali (2018). Briefly, EEG recordings are obtained using a Biosemi Active Two 128-electrode array, initially sampled at 2048 Hz. Each of the 20 scenes is presented to each subject one time in a random order, and listeners are asked to ignore these scenes playing in the background. Concurrently, subjects are presented with a sequence of tones and perform an amplitude modulation detection task. The neural data relevant to the modulation task is not relevant to the current study and is not presented here. It is discussed in the study by Huang and Elhilali (2018).

Electroencephalography signals are analyzed using FieldTrip (Oostenveld et al., 2011) and EEGLab (Delorme and Makeig, 2004) analysis tools. Data are demeaned and detrended, and then resampled at 256 Hz. Power line energy is removed using the Cleanline MATLAB plugin (Mullen, 2012). EEG data are then re-referenced using a common average reference, and eyeblink artifacts are removed using independent component analysis (ICA).

Following these preprocessing steps, energy at various frequency bands is isolated using a Fourier transform over sliding windows (length 1 s, step size 100 ms), and then averaged across the frequencies in a specific band. Six such frequency bands are used in the analysis to follow: Delta (1–4 Hz), Theta (4–7 Hz), Alpha (8–15 Hz), Beta (15–30 Hz), Gamma (30–50 Hz), and High Gamma (70–110 Hz). Next, band energy is z-score normalized within each channel. Band activity is analyzed both on a per-electrode basis and also by averaging activity across groups of electrodes. In addition to a grand average across all 128 electrodes, analysis is also performed by averaging activity in frontal electrodes (21 electrodes near Fz) and central electrodes (23 electrodes near Cz) as defined in Shuai and Elhilali (2014).

### Deep Neural Network

**FIGURE 1:**
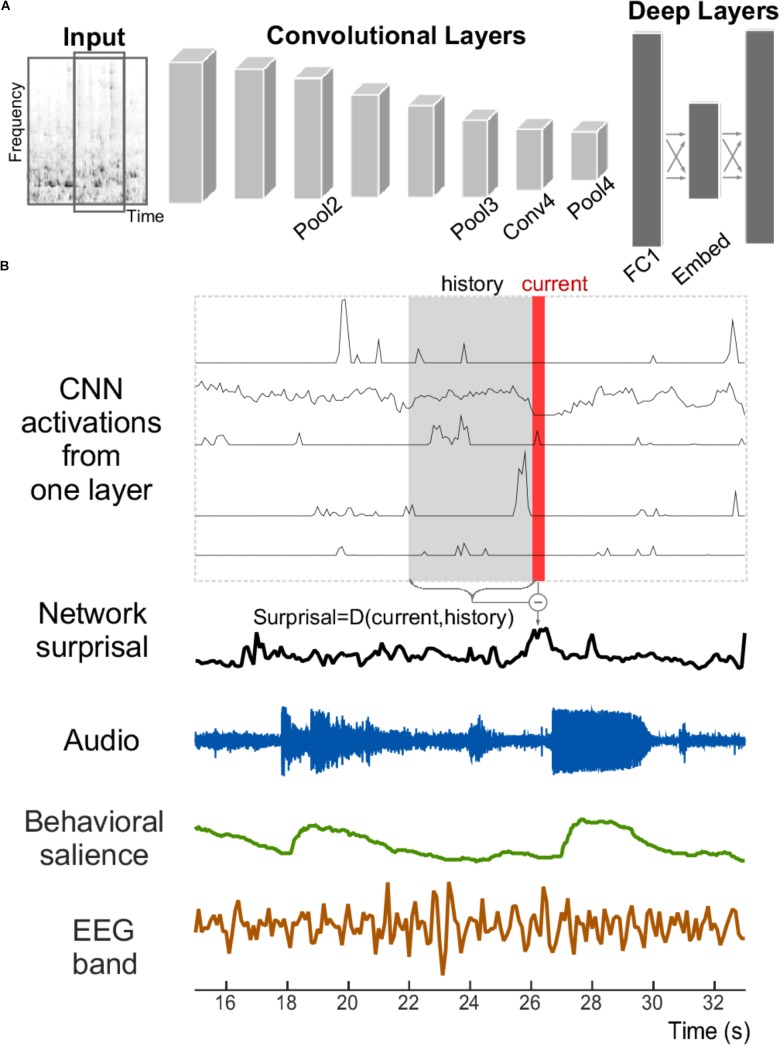
Structure of the convolutional neural network and signals analyzed. **(A)** The convolutional neural network receives the time–frequency spectrogram of an audio signal as input. It is composed of convolutional and pooling layers in an alternating fashion, followed by fully connected layers. **(B)** An example section of an acoustic stimulus (labeled Audio); along with corresponding neural network activity from five example units within one layer of the CNN. A network surprisal measure is then computed as the Euclidian distance between the current activity of the network nodes at that layer (shown in red) against the activity in a previous window (shown in gray with label “History”). Measures of behavioral salience by human listeners (in green) and cortical activity recorded by EEG (in brown) are also analyzed.

A neural network is used in the current study to explore its relationship with salience judgments based on acoustic analysis, behavioral measures, and neural EEG responses (**Figure 1**). The network structure like VGG follows network E presented by Simonyan and Zisserman (2015), with modifications made by Hershey et al. (2017) and Jansen et al. (2017). Briefly, the network staggers convolutional and pooling layers. It contains four convolutional layers, each with relatively small 3 × 3 receptive fields. After each convolutional layer, a spatial pooling layer reduces the number of units by taking maximums over non-overlapping 2 × 2 windows. Next, two fully connected layers then reduce the dimensionality further before the final prediction layer. **Table 1** lists the layers of the network along with their respective dimensionalities. Due to dimensionality constraints, only the layers shown in bold are used in this analysis and reported here, without any expected loss of generality about the results.

**Table 1:** Dimensions of the input and each layer of the neural network.

Layer type	Abbreviation	Dimensions	Total number of outputs
Input spectrogram		96 × 64	16,384
Convolutional layer	Conv1	96 × 64 × 64	393,216
Pooling layer	Pool1	48 × 32 × 64	98,304
Convolutional layer	Conv2	48 × 32 × 128	196,608
**Pooling layer**	**Pool2**	**24 × 16 × 128**	**49,152**
Convolutional layer	Conv3	24 × 16 × 256	98,304
**Pooling layer**	**Pool3**	**12 × 8 × 256**	**24,576**
**Convolutional layer**	**Conv4**	**12 × 8 × 512**	**49,152**
**Pooling layer**	**Pool4**	**6 × 4 × 512**	**12,288**
**Fully connected layer**	**FC1**	**4096**	**4096**
**Fully connected layer**	**Embed**	**128**	**128**
Output layer/predictions	Predic	4923	4923


*Bold text indicates which layers are used in the analysis.*

Our CNN was trained on the audio from a 4923 class video-classification problem that eventually became the YouTube-8M challenge (Abu-El-Haija et al., 2016). This dataset includes 8 million videos totaling around 500,000 h of audio, and is available online (Abu-El-Haija, 2017). As in the study by Hershey et al. (2017), the audio from each video was divided into 960 ms frames, each mapped onto a time–frequency spectrogram (25 ms window, 10 ms step size, 64 mel-spaced frequency bins). This spectrogram served as the input to the neural network. For training purposes, ground truth labels from each video were automatically generated and every frame within that video was assigned the same set of labels. Each video could have any number of labels, with an average of around five per video, and 4923 distinct labels in total. The labels ranged from very general to very specific. The most general category labels (such as arts and entertainment, games, autos/vehicles, and sports) were applied to roughly 10–20% of the training videos. The most specific labels (such as classical ballet, rain gutter, injury, and FIFA Street) applied only to 0.0001–0.001% of the videos. The network was trained to optimize classification performance over the ground truth labels. The network’s classification performance nearly matches that of the Inception DNN model, which was found to show the best results in Hershey et al. (2017), in terms of equal error rate and average precision. Details about the evaluation process can be found in Jansen et al. (2017).

### Network Surprisal

We defined change in the activation patterns within a layer of the CNN as “*network surprisal*” (this definition is unrelated to other surprisal analyses that employ information theory or principles of thermodynamics to characterize system dynamics, often used in physics, chemistry, and other disciplines). It represents an estimate of variability in the response pattern across all nodes of a given layer in the network and as such quantifies how congruent or surprising activity at a given moment is relative to preceding activity (**Figure 1B**). In this study, it is computed by taking the Euclidean distance between the activity in a layer at a given time bin (labeled “Current” in red in **Figure 1B**) vs. the average activation in that layer across the previous four seconds (labeled “History” in gray in **Figure 1B**). Thus, a constant pattern of activity would result in a low level of surprisal, while a fluctuation in that pattern over multiple seconds would result in a higher level of surprisal. This measure corresponds structurally to the definition of semantic dissimilarity by Broderick et al. (2018), although it utilizes Euclidean distance as a common metric for evaluating dissimilarity in neural network activity (Krizhevsky et al., 2012; Parkhi et al., 2015). This surprisal feature tracks changes in the scene as it evolves over time by incorporating elements of the acoustic history into its calculation.

### Correlation Analyses

The audio, EEG, and CNN data have all been reduced to low-dimensional features. The audio is represented by 10 different acoustic measures, while the 128 channel EEG measurements are summarized by the energy in six different frequency bands, and the multi-channel outputs from the six different layers of the CNN are summarized by the surprisal measure. We next examine correlation between these metrics and the neural network activations.

Each layer of the neural network is compared to behavioral salience, basic acoustic features, and energy in EEG frequency bands using normalized cross correlation. All signals are resampled to the same sampling rate of 10 Hz, and the first 2 s of each scene are removed to avoid the effects of the trial onset. Scenes that are longer than 120 s are shortened to that length. All signals are high-pass filtered with a cutoff frequency of 1/30 Hz to remove overall trends, and then low-pass filtered at 1/6 Hz to remove noise at higher frequencies. Both filters are fourth-order Butterworth filters. The low-pass cutoff frequency is chosen empirically to match the slow movements in the salience signal. Despite the low cutoff frequency, no observable ringing artifacts are noted. Adjusting signal duration to examine any filtering artifacts at the onset of the signal yields quantitively similar results as reported in this paper.

After these pre-processing steps, we compute the normalized cross-correlation between network surprisal and the other continuous (acoustic and neural) signals with a maximum delay time of -3 to +3 s. The normalized correlation is defined as a sliding dot-product of these two signals normalized by the product of their standard deviation (Rao Yarlagadda, 2010). The highest correlation coefficient within a ± 3 s window is selected as the correlation between network surprisal and each of the corresponding signals.

The behavioral responses reflect onsets of salient events (peaks in the slope of the salience curve) and are discrete in time. CNN surprisal activity is compared to behavioral salience in windows surrounding salient events, extending from 3 s before to 3 s after each event. These windows are used to compare correlations for subsets of events, such as for a single category of events. Quantitatively similar results are obtained when using the whole salience curve instead of windows surrounding all salient events. The correlation coefficient between behavioral salience and neural surprisal vectors is taken in these windows. For this analysis, the behavioral salience signal is delayed by a fixed time of 1.4 s. A shift is necessary to reflect the delay in motor response required from the behavioral task to report salience. Here, a shift of 1.4 s is empirically determined to correspond to the maximum cross correlation for a majority of the network layers. A fixed delay is used for this case for greater consistency when comparing across different conditions.

To complement the correlation analysis described above, we also examine the cumulative contribution of different CNN layers by assessing the cumulative variance explained by combining activation of consecutive layers. This variance is quantified using a linear regression that uses behavioral salience as the dependent variable and network surprisal from individual layers as independent variables (Weisberg, 2005). Consecutive linear regressions with each layer individually are performed starting with lower layers and continuing to higher layers of the network. After each linear regression, the cumulative variance explained is defined as 1 minus the variance of the residual divided by the variance of the original salience curve (i.e., 1 minus the fraction of variance explained). Then, the residual is used as the independent variable for regression with the next layer. To generate a baseline level of improvement by increasing the number of layers, this linear regression procedure is repeated after replacing all values in layers after the first with numbers generated randomly from a normal distribution (mean 0, variance 1).

### Event Prediction

Prediction of salient events is performed by dividing the scene into overlapping time bins (2 s bin size, 0.5 s step size) and then using linear discriminant analysis (LDA; Duda et al., 2000). Each time bin is assigned a label of +1 if a salient event occurred within its respective time frame and a label of 0 otherwise. Network surprisal and the slopes of acoustic features are used to predict salient event using an LDA classifier. The slope of an acoustic feature is calculated by first taking the derivative of the signal, and then smoothing it with three iterations of an equally weighted moving average (Huang and Elhilali, 2017). This smoothing process is selected empirically to balance removal of higher frequency without discarding potential events. As with the previous event-based analysis, these signals are time-aligned by maximizing their correlation with behavioral salience. Each feature is averaged within each time bin, and LDA classification is performed using fivefold cross validation to avoid overfitting (Izenman, 2013). Finally, a threshold is applied to the LDA scores at varying levels to obtain a receiver operating characteristic (ROC) curve (Fawcett, 2006).

## Results

This section describes the correlation between the six different layers of the CNN vs. the 10 acoustic features, salience as measured by a behavioral task, and energy in six different frequency bands from the EEG data.

### Comparison to Basic Acoustic Features

**FIGURE 2:**
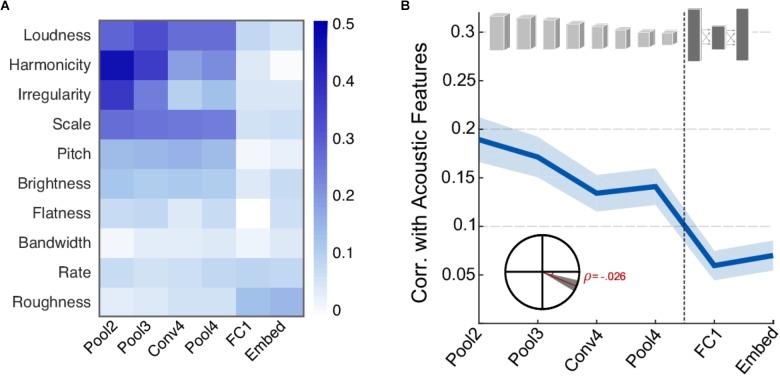
Correlation between neural network activity and acoustic features. **(A)** Correlation coefficients between individual acoustic features and layers of the neural network. Loudness, harmonicity, irregularity, scale, and pitch are the most strongly correlated features overall. **(B)** Average correlation across acoustic features and layers of the neural network. Shaded area depicts ±1 standard error of the mean (SEM). Inset shows the slope of the trend line fitted with a linear regression. The shaded area depicts 99% confidence intervals of the slope.

First, we examine the correspondence between activity in different neural network layers and the acoustic features extracted from each of the scenes. **Figure 2A** shows the correlation coefficient between each acoustic feature and the activity of individual CNN layers. Overall, the correlation pattern reveals stronger values in the four earliest layers (convolutional and pooling) compared the deep layers in the network (fully connected and embedding). This difference is more pronounced in features of a more spectral nature such as spectral irregularity, frequency modulation, harmonicity, and loudness, suggesting that such features may play an important role in informing the network about sound classification during the training of the network. Clearly, not all acoustic features show this strong correlation or any notable correlation. In fact, roughness and rate are basic acoustic measures that show slightly higher correlation in deeper layers relative to earlier layers. **Figure 2B** summarizes the average correlation across all basic acoustic features used in this study as a function of network layer. The trend reveals a clear drop in correlation, indicating that the activity in deeper layers is more removed from the acoustic profile of the scenes. **Figure 2B** inset depicts a statistical analysis of this drop, with slope = -0.026, *t*(1198) = -5.8, p = 7.6 × 10^-9^.

**FIGURE 3:**
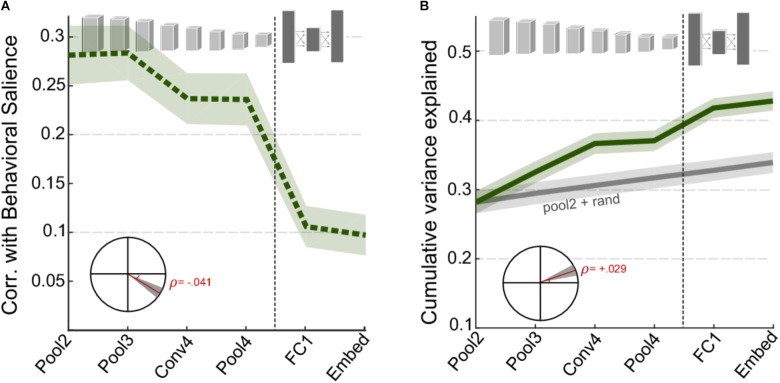
CNN surprisal and behavioral salience. **(A)** Correlation between CNN activity and behavioral salience. **(B)** Cumulative variance explained after including successive layers of the CNN. The gray line shows a baseline level of improvement estimated by using values drawn randomly from a normal distribution for all layers beyond Pool2. For both panels, shaded areas depict ±1 SEM. Insets show the slope of the trend line fitted with a linear regression, with shaded areas depicting 99% confidence intervals of the slope.

Next, we examine the correspondence between activations in the CNN layers and the behavioral judgments of salience as reported by human listeners. **Figure 3A** shows the correlation between behavioral salience and network surprisal across individual layers of the network, taken in windows around salient events (events being local maxima in the derivative of salience, see section “Materials and Methods”). As noted with the basic acoustic features (**Figure 2**), correlation is higher for the earlier layers of the CNN and lower for the later layers. A statistical analysis of the change in correlation across layers reveals a significant slope of -0.041, *t*(1360) = -6.8, *p* = 2.1 **×** 10^-11^ (**Figure 3A**, inset). However, although the correlation for individual deeper network layers is relatively poor, an analysis of their complementary information suggests additional independent contributions of each layer. In fact, the cumulative variance explained as one goes deeper into the network shows significantly improved correlation between superficial and deep layers (**Figure 3B**), with a correlation slope of 0.029, *t*(1360) = 6.6, *p* = 5 × 10^-11^.

**FIGURE 4:**
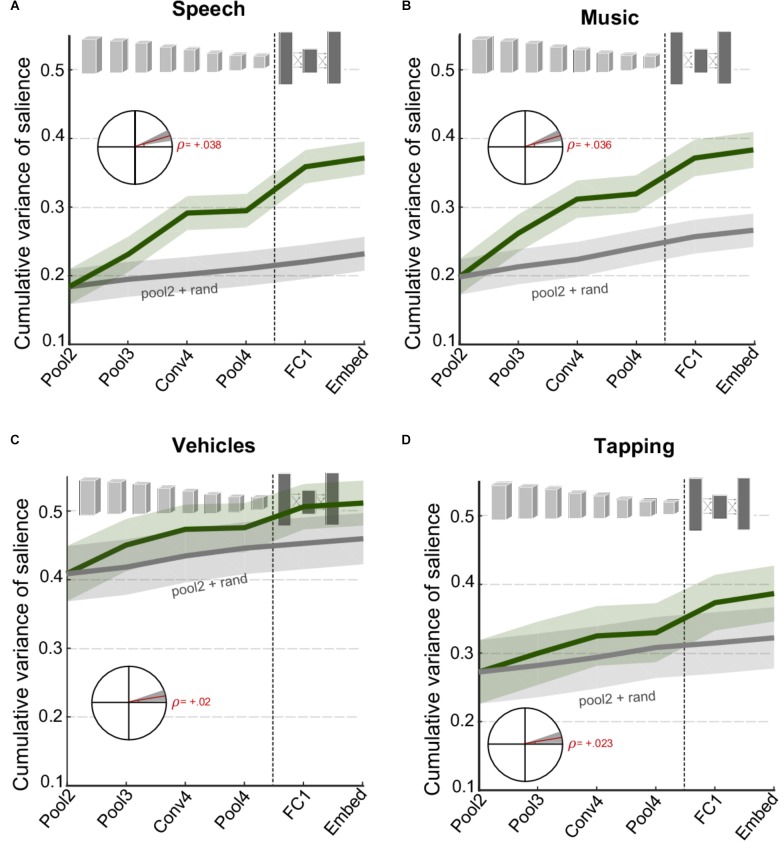
Cumulative variance explained after including successive layers of the CNN for specific categories of events **(A)** speech events, **(B)** music events, **(C)** vehicle events, and **(D)** tapping/striking events. The gray line shows a baseline level of improvement estimated by using values drawn randomly from a normal distribution for all layers beyond Pool2. For all panels, shaded areas depict ±1 SEM. Insets show the slope of the trend line fitted with linear regression, with shaded areas depicting 99% confidence intervals of the slope.

While **Figure 3** looks at complementary information of different network layers in explaining behavioral judgments of salience *on average*, one can look explicitly at specific categories of events and examine changes in information across CNN layers. **Figure 4** contrasts the cumulative variance explained for four classes of events that were identified manually in the database (see section “Materials and Methods”). The figure compares cumulative variance of behavioral salience explained by the network for speech, music, vehicle, and tapping events. The figure shows that speech and music-related events are better explained with the inclusion of deeper later layers [speech: *t*(280) = 5.2, *p* = 3.2 × 10^-7^; music: *t*(340) = 5.7, *p* = 3.3 × 10^-08^]. In contrast, events from the devices/vehicles and tapping categories are well explained by only the first few peripheral layers of the network, with little benefit provided by deeper layers [device: *t*(262) = 1.8, *p* = 0.069; tapping: *t*(166) = 2.2, *p* = 0.028]. Results for other vocalizations closely match those of the vehicle category (data not shown), *t*(196) = 2.2, *p* = 0.033. Overall, the figure highlights that contribution of different CNN layers to perceived salience of different scenes does vary drastically depending on semantic meaning and show varying degrees of complementarity between the acoustic front-end representation and the semantic deeper representations.

**FIGURE 5:**
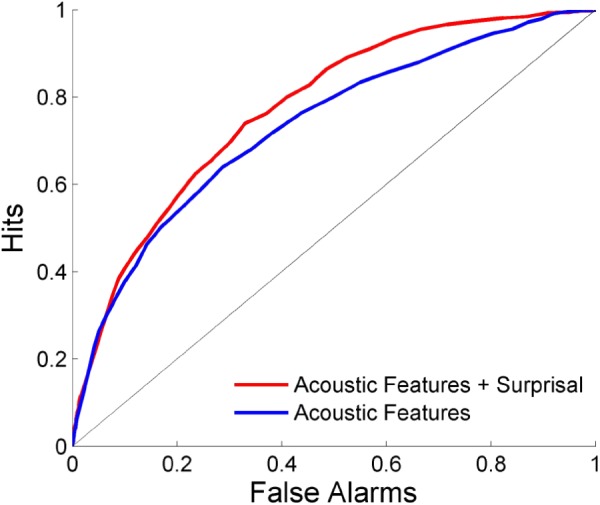
Event prediction performance. Predictions are made using LDA on overlapping time bins across scenes. The area under the ROC curve is 0.775 with a combination of acoustic features and surprisal, while it reaches only 0.734 with acoustic features alone.

The ability to predict where salient events occur is shown in **Figure 5**. Each scene is separated into overlapping time bins which are labeled based on whether or not an event occurred during that time frame. LDA is then performed using either a combination of acoustics and network surprisal, or the acoustic features alone. The prediction is improved through the inclusion of information from the neural network, with an area under the ROC curve of 0.734 when using only the acoustic features compared to an area of 0.775 after incorporating network surprisal. This increase in performance indicates that changes in network activity make a contribution to the salience prediction that is not fully captured by the acoustic representation.

**FIGURE 6:**
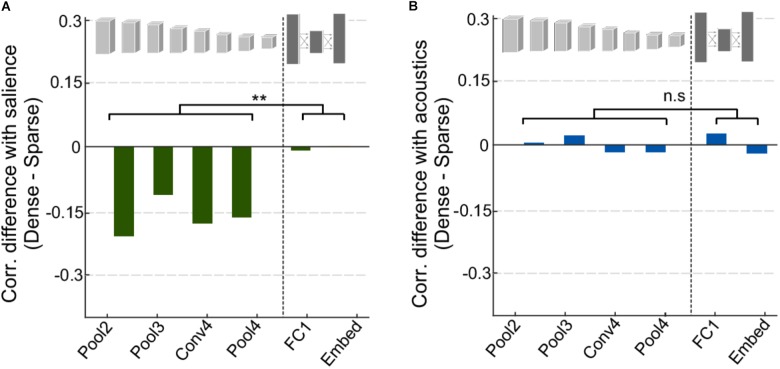
Analysis of dense vs. sparse scenes. **(A)** Difference in correlation between salience and CNN activity for dense and sparse scenes. Negative values indicate that salience in sparse scenes was more highly correlated with CNN activity. **(B)** Difference in correlation between acoustic features and CNN activity for dense and sparse scenes.

One of the key distinctions between the different event categories analyzed in **Figure 4** is not only the characteristics of the events themselves but also the context in which these events are typically present. On the one hand, speech scenes tend to have ongoing activity and dynamic backgrounds against which salient events stand out; while vehicle scenes tend to be rather sparse with few notable events standing out as salient. An analysis contrasting sparse vs. dense scenes in our entire dataset (see section “Materials and Methods”) shows a compelling difference between the correlations of acoustic salience for dense scenes and for sparse scenes especially in the convolutional layers (**Figure 6A**). This difference is statistically significant when comparing the mean correlation for early vs. deep layers, *t*(4) = -5.4, *p* = 0.0057. On the other hand, the network’s activation in response to acoustic profiles in the scenes do not show any distinction between sparse and dense scenes and across early and deep layers (**Figure 6B**), *t*(4) = -0.24, *p* = 0.82.

**FIGURE 7:**
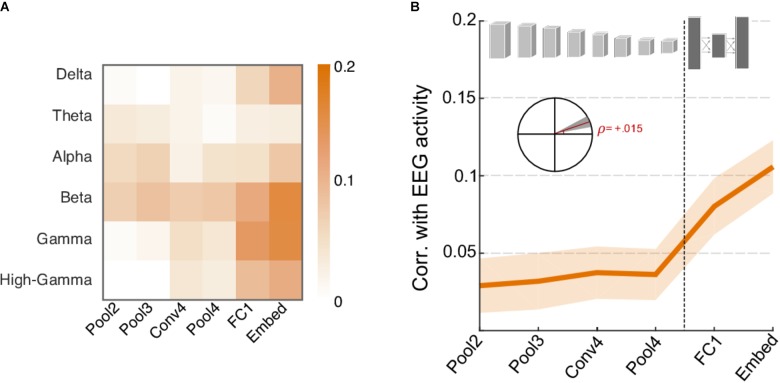
Correlation between neural network activity and energy in EEG frequency bands. **(A)** Correlation coefficients between individual EEG frequency bands and layers of the neural network. Gamma, Beta, and High-Gamma frequency bands are the most strongly correlated bands overall. **(B)** Average correlation across EEG activity and layers of the neural network. Shaded area depicts ±1 SEM. Inset shows the slope of the trend line fitted with linear regression, with a shaded area depicting the 99% confidence interval of the slope.

Finally, we examine the contrast between neural responses recorded using EEG and CNN activations. As shown in **Figure 7**, energy in many frequency bands of the neural signal shows stronger correlation with activity in higher levels of the CNN rather than lower layers and follows an opposite trend to that of acoustic features. **Figure 7A** shows the correlation between network activity and individual EEG frequency bands and shows a notable increase in correlation for higher frequency bands (Delta, Beta, Gamma, and High Gamma). The Theta and Alpha bands appear to follow a somewhat opposite trend, though their overall correlation values are rather small. **Figure 7B** summarizes the average correlation trend across all frequency bands, with slope = 0.015, *t*(718) = 3.6, *p* = 3.2 × 10^-4^). It is worth noting the average correlation between CNN activity and EEG responses is rather small overall (between 0 and 0.1) but still significantly higher than 0, *t*(719) = 7.4, *p* = 4.5 × 10^-13^. The increasing trend provides further support to the notion that higher frequency neural oscillations are mostly aligned with increasingly complex feature and semantic representations crucial for object recognition in higher cortical areas, and correspondingly in deeper layers of the CNN (Kuzovkin et al., 2017).

**FIGURE 8:**
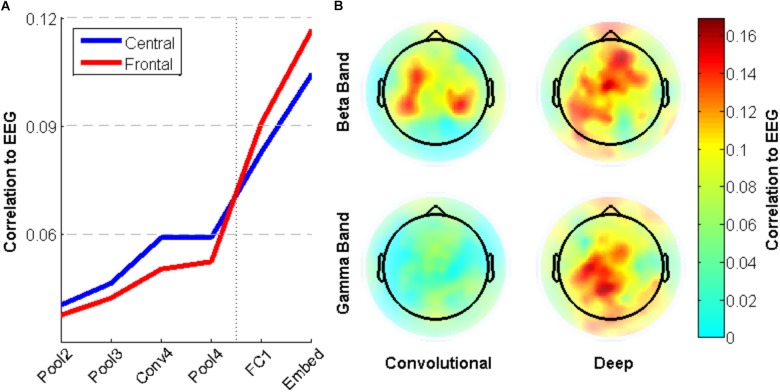
Correlation between neural network activity and energy in EEG frequency bands for specific electrodes. **(A)** Average correlation between electrode activity across frequency bands for electrodes in central (near Cz) and frontal (near Fz) regions. **(B)** Correlation between beta/gamma band activity for individual electrodes and convolutional/deep layers of the neural network.

To explore the brain regions that are most closely related to the CNN activity, individual electrode activities are also correlated with surprisal. **Figure 8A** shows a small difference between neural activity in Central and Frontal areas, with the former having relatively higher correlation with early layers and the latter having higher correlation with deep layers. This trend is not statistically significant, however. **Figure 8B** shows the pattern across electrodes of these correlations values for the beta and gamma bands. Activity in the Beta band is most correlated to the convolutional layers of the CNN for central electrodes near C3 and C4, while it is most correlated to the deep layers for frontal electrodes near Fz. In contrast, Gamma band activity shows little correlation with the early layers of the CNN, but more closely matches activation in deep layers for electrodes near Cz.

## Discussion

Recent work on deep learning models provides evidence of strong parallels between the increasing complexity of signal representation in these artificial networks and the intricate sensory transformations in sensory biological systems that map incoming stimuli onto object-level representations (Yamins et al., 2014; Guclu and van Gerven, 2015; Cichy et al., 2016). The current study leverages the complex hierarchy afforded by CNNs trained on audio classification to explore parallels between network activation and auditory salience in natural sounds measured through a variety of modalities. The analysis examines the complementary contribution of various layers in a CNN architecture and draws a number of key observations from three types of signals: acoustic, behavioral, and neural profiles.

First, as expected, the earlier layers in the CNN network mostly reflect the acoustic characteristics of a complex soundscape. The association of acoustic features with CNN activation decreases in correlation as the signal propagates deeper into the network. The acoustic features that are most clearly reflected with higher fidelity are mostly spectral, and include harmonicity, frequency modulation, and spectral irregularity, along with loudness which directly modulates overall signal levels. It is important to remember that the CNN network used in the current work is trained for audio classification and employs a rather fine-resolution spectrogram at its input computed with 25 ms bins over frames of about 1 s. As such, it is not surprising to expect a strong correlation between spectral features in the input and early representations of the peripheral layers of the CNN network (Dai et al., 2017; Lee et al., 2017; Wang et al., 2017). Interestingly, two features that are temporal in nature, namely, rate and most prominently roughness, show a somewhat opposite trend with a mildly increased correlation with deeper CNN layers. Both these acoustic measures quantify the degree of amplitude modulations in the signal over longer time scales of tens to hundreds of milliseconds, and we can speculate that such measures would involve longer integration levels that are more emblematic of deeper layers in the network that pool across various localized receptive fields. The distributed activation of CNN layers reflecting various acoustic features supports previous accounts of hierarchical neural structures in auditory cortex that combine low-level and object-level representations extending beyond the direct physical attributes of the scenes (Formisano et al., 2008; Staeren et al., 2009). This distributed network suggests an intricate, multi-region circuitry underlying the computation of sound salience in the auditory system, much in line with reported underpinnings of visual salience circuits in the brain (Veale et al., 2017).

Second, the results show a strong correlation between peripheral layers of the CNN and behavioral reports of salience. This trend is not surprising given the important role acoustic characteristics of the signal play in determining the salience of its events (Kaya and Elhilali, 2014; Kim et al., 2014; Huang and Elhilali, 2017). This view is then complemented by the analysis of cumulative variance explained by gradually incorporating activation of deeper layers in the neural network. **Figure 3** clearly shows that information extracted in later layers of the network supplements activation in earlier layers and offers an improved account of auditory salience. This increase is maintained even at the level of the fully connected layers suggesting a complementary contribution of low-level and category-level cues in guiding auditory salience. This observation is further reinforced by focusing on salience of specific sound categories. In certain cases that are more typical of sparse settings with prominent events such as tapping or vehicle sounds, it appears that the low-level acoustic features are the main determinants of auditory salience with little contribution from semantic-level information. In contrast, events in the midst of a speech utterance or a musical performance appear to have a significant increase in variance explained by incorporating all CNN layers (**Figure 4**). The complementary nature of peripheral and object-level cues is clearly more prominent when taking into account the scene context, by contrasting denser, busy scenes with quieter environments with occasional, prominent events. Dense settings typically do not have as many conspicuous clear changes in acoustic information across time, and as a result, they seem to require more semantic-level information to complement information from acoustic features for a complete account of auditory salience.

Third, the CNN layer activation shows an opposite correlation trend with neural oscillation measured by EEG. In particular, the deeper layers of the neural network have higher correlation with activity in the higher frequency bands (beta, gamma, and high gamma bands). Synchronous activity in the Gamma band has been shown to be associated with object representation (Rodriguez et al., 1999; Bertrand and Tallon-Baudry, 2000), which would be directly related to the audio classification task. Activity in both the Gamma and Beta bands has also been linked to hearing novel stimuli (Haenschel et al., 2000). Moreover, Gamma band activity is known to be strongly modulated by attention (Tiitinen et al., 1993; Müller et al., 2000; Doesburg et al., 2008), which further reinforces the relationship between object category and salience.

In particular, the CNN activation patterns of the deep layers correlate most strongly with neural oscillations in frontal areas of the brain. This finding expands on the recent work by Kell et al. (2018), which found that activation patterns within intermediate layers of their CNN were the best at predicting activity in the auditory cortex. It stands to reason that later layers of the network would correspond more to higher level brain regions, which may play a role in attention and object recognition.

Overall, all three metrics used in the current study offer different accounts of conspicuity of sound events in natural soundscapes. By contrasting these signals against activations in a convolutional DNN trained for audio recognition, we are able to assess the intricate granularity of information that drives auditory salience in everyday soundscapes. The complexity stems from the complementary role of cues along the continuum from low-level acoustic representation to coherent object-level embeddings. Interestingly, the contribution of these different transformations does not uniformly impact auditory salience for all scenes. The results reveal that the context of the scene plays a crucial role in determining the influence of acoustics or semantics or possibly transformations in between. It is worth noting that the measure of surprisal used here is but one way to characterize surprise. Looking at changes in a representation compared to the average of the last few seconds is simple and proves to be effective. However, different ways to capture the context, perhaps including fitting the data to a multimodal Gaussian mixture model, as well as different time scales should be investigated.

Further complicating the interaction with context effects is the fact that certain acoustic features should not be construed as simple transformation of the acoustic waveform or the auditory spectrogram. For instance, a measure such as roughness appears to be less correlated with lower layers of the CNN. This difference suggests that acoustic roughness may not be as readily extracted from the signal as the other acoustic measures by the neural network, but it is nonetheless important for audio classification and correlates strongly with perception of auditory salience (Arnal et al., 2015).

One limitation of the CNN structure is that it only transmits information between layers in the forward direction, while biological neural systems incorporate both feedforward and feedback connections. Feedback connections are particularly important in studies of attention because salience (bottom-up attention) can be modified by top-down attention. This study uses behavioral and physiological data that were collected in such a way that the influence of top-down activity was limited; however, a complete description of auditory attention would need to incorporate such factors. An example of a feedback CNN that seeks to account for top-down attention can be found in Cao et al. (2015).

It is not surprising that our limited understanding of the complex interplay between acoustic profiles and semantic representations has impeded development of efficient models of auditory salience that can explain behavioral judgments, especially in natural, unconstrained soundscapes. So far, most accounts have focused on incorporating relevant acoustic cues that range in complexity from simple spectrographic representation to explicit representation of pitch, timbre, or spectro-temporal modulation (Duangudom and Anderson, 2007; Kalinli and Narayanan, 2007; Tsuchida and Cottrell, 2012; Kaya and Elhilali, 2014). However, as highlighted by the present study, it appears that a complementary role of intricate acoustic analysis (akin to that achieved from the complex architecture of convolutional layers in the current CNN) as well as auditory object representations will be necessary to not only account for contextual information about the scene but may determine the salience of a sound event depending on its category, sometimes regardless of its acoustic attributes.

## Ethics Statement

This study was carried out in accordance with the recommendations of the Belmont Report and the Homewood Institutional Review Board at the Johns Hopkins University. The protocol was approved by the Homewood Institutional Review Board. All subjects gave written informed consent in accordance with the Declaration of Helsinki.

## Author Contributions

All authors contributed to the conception and design of the study, led by ME. NH collected behavioral and EEG data and conducted statistical analysis. MS performed the neural network computation. All authors contributed to manuscript write up and read and approved the submitted version.

## Conflict of Interest Statement

MS is employed by Google AI. The remaining authors declare that the research was conducted in the absence of any commercial or financial relationships that could be construed as a potential conflict of interest.
